# Evaluation of different ^89^Zr-labeled synthons for direct labeling and tracking of white blood cells and stem cells in healthy athymic mice

**DOI:** 10.1038/s41598-022-19953-4

**Published:** 2022-09-19

**Authors:** Aditya Bansal, Shalini Sharma, Benedikt Klasen, Frank Rösch, Mukesh K. Pandey

**Affiliations:** 1 Division of Nuclear Medicine, Department of Radiology, Mayo Clinic, Rochester, MN 55906 USA; 2 Department of Chemistry-TRIGA Site, Johannes Gutenberg University, Mainz, Germany

**Keywords:** Biological techniques, Stem cells, Chemistry

## Abstract

Cell based therapies are evolving as an effective new approach to treat various diseases. To understand the safety, efficacy, and mechanism of action of cell-based therapies, it is imperative to follow their biodistribution noninvasively. Positron-emission-tomography (PET)-based non-invasive imaging of cell trafficking offers such a potential. Herein, we evaluated and compared three different ready-to-use direct cell radiolabeling synthons, [^89^Zr]Zr-DFO-Bn-NCS, [^89^Zr]Zr-Hy_3_ADA^5^-NCS, and [^89^Zr]Zr-Hy_3_ADA^5^-SA for PET imaging-based trafficking of white blood cells (WBCs) and stem cells (SCs) up to 7 days in athymic nude mice. We compared the degree of ^89^Zr complexation and percentage of cell radiolabeling efficiencies with each. All three synthons, [^89^Zr]Zr-DFO-Bn-NCS, [^89^Zr]Zr-Hy_3_ADA^5^-NCS, and [^89^Zr]Zr-Hy_3_ADA^5^-SA, were successfully prepared, and used for radiolabeling of WBCs and SCs. The highest cell radiolabeling yield was found for [^89^Zr]Zr-DFO-Bn-NCS, followed by [^89^Zr]Zr-Hy_3_ADA^5^-NCS, and [^89^Zr]Zr-Hy_3_ADA^5^-SA. In terms of biodistribution, WBCs radiolabeled with [^89^Zr]Zr-DFO-Bn-NCS or [^89^Zr]Zr-Hy_3_ADA^5^-NCS, were primarily accumulated in liver and spleen, whereas SCs radiolabeled with [^89^Zr]Zr-DFO-Bn-NCS or [^89^Zr]Zr-Hy_3_ADA^5^-NCS were found in lung, liver and spleen. A high bone uptake was observed for both WBCs and SCs radiolabeled with [^89^Zr]Zr-Hy_3_ADA^5^-SA, suggesting in-vivo instability of [^89^Zr]Zr-Hy_3_ADA^5^-SA synthon. This study offers an appropriate selection of ready-to-use radiolabeling synthons for noninvasive trafficking of WBCs, SCs and other cell-based therapies.

## Introduction

Safety and efficacy are the two main pillars of any therapeutics and cell-based therapies and imaging are no exception. Not much is known, to effectively assess the biodistribution, clearance and efficacy of cell-based therapies due to the absence of an appropriate noninvasive imaging tool. In vivo cell tracking could provide information about distribution, localization, and clearance of various cell-based therapies including immune cells (CAR-T cells), stem cells and hepatocytes post-administration in the body. There are various non-invasive molecular imaging modalities that could be employed to track cell based therapies including optical imaging via fluorescence imaging (FLI)^1,2^, bioluminescence imaging (BLI)^3,4^, and ultrasound-guided photoacoustic imaging (PA)^5–7^. Radiology imaging including magnetic resonance imaging (MRI)^8–10^, computed tomography (CT)^11–13^, and nuclear medicine imaging such as positron emission tomography (PET)^14–18^ and single photon emission computed tomography (SPECT)^19,20^, could also be employed to effectively measure the distribution, localization, and clearance of various cell-based therapies over time and to shed light on safety and efficacy.

Among various imaging modalities, optical imaging modalities are restricted to small animals due to limited tissue penetration (1–2 mm) in humans. MRI and CT provide high resolution anatomical information, but have low sensitivity in both animals and humans. Both PET and SPECT are advantageous over other techniques and are often integrated with CT and MRI. The PET/CT or SPECT/CT or PET/MRI provide quantitative and temporal distribution of immune and stem cells in animals and patients with no limitation of tissue penetration due to high energy gammas^21–24^.

Cells can be radiolabeled either directly or indirectly^25^. Direct cell radiolabeling consists of ex-vivo radiolabeling of cells prior to their administration into body followed by short-term (< 7 days) in vivo tracking of these radiolabeled cells. The potential limitation of the direct cell labeling approach is the short-term tracking capability due to decay of the radioactivity over time and or efflux of radiotracer or instability of the labeled radioactive tag over time. On the other hand, the indirect cell radiolabeling method is based on transfection of a reporter gene (e.g., sodium iodide symporter (NIS)^26^, simplex herpes virus type -1 thymidine kinase (HSV1-tk) etc.) in the cells that selectively takes up the respective radioactive reporter probe in the cells upon exposure to its respective reporter probe. If the administered cells keep expressing reporter protein after administration, then repeated systemic administration of its reporter probe allows long term-visualization of administered cells. Although the indirect cell labeling approach allows long term visualization of administered cells, genetic modification for cell labeling remains a regulatory hurdle.

Cell radiolabeling using a direct radiolabeling approach with various SPECT radiopharmaceuticals such as [^99m^Tc]Tc-HMPAO (t_1/2_ = 6.01 h)^27–29^, and [^111^In]In-oxine (t_1/2_ = 68.2 h)^30–33^ have been used to track leukocytes for infection and inflammation imaging over the past four decades. SPECT is a powerful clinical imaging tool with lower usage cost than PET since an onsite cyclotron is not needed. PET, however, has many advantages over SPECT including two to threefold higher sensitivity, superior spatial resolution in the clinical setting, and with its quantitative nature it is a preferred imaging modality for tracking a single cell or small number of administered radiolabeled cells with more precise quantification and hence, requires lower radiation exposure^34^. Examples of commercial PET probes used to label cells include [^18^F]FDG (t_1/2_ = 109.7 min, β^+^  = 97%)^35^, [^64^Cu]Cu-PTSM (t_1/2_ = 12.7 h, β^+^  = 17.9% )^36^ and [^68^ Ga]Ga-oxine (t_1/2_ = 68 min, β^+^  = 88.8%)^37,38^.

Recently, among various PET radioisotopes, zirconium-89 (β^+^  = 22.3%) is gaining popularity for cell tracking due to its well established cyclotron-mediated production, longer half-life of 3.27 days and low average positron energy (E_β+_ = 0.395 MeV). This enables monitoring of radiolabeled cells up to 3-weeks, either through direct cell labeling (also called non-specific cell labeling agents)^39,40^ or indirect labeling mediated through antibodies^41,42^, peptides^43^, proteins^44^ and nanoparticles^45–47^.

Various chelators used for the radiolabeling of cells with ^89^Zr are tropolone, malonate, hydroxamates, and oxine (8-hydroxyquinoline). Among these, oxine forms a lipophilic complex with ^89^Zr and enters the cells passively. To date, [^89^Zr]Zr-oxine is a commonly used radiotracer to label various cells including tumor cell lines^48,49^, bone marrow cells^50,51^, T cells^52^, NK cells^53^, white blood cells (WBCs)^54^, stem cells (SCs)^55^ and leukocytes^56^. However, efflux of ^89^Zr from cells labeled with [^89^Zr]Zr-oxine remains a challenge. Recently, Friberger et al. reported a one-step clinically translatable method of synthesis of [^89^Zr]Zr-oxine with a cell labeling efficiency of 61–68% with human decidual stromal cells (hDSCs), bone marrow-derived macrophages (rMac) and human peripheral blood mononuclear cells (hPBMCs). However, a 29–38% apparent efflux of ^89^Zr from the labeled cells raised a further concern of radiotoxicity and non-specificity of the signal^57^.

Besides [^89^Zr]Zr-oxine, the other reported method of cell labeling was covalent attachment of radiolabeled [^89^Zr]Zr-DFO-Bn-NCS complex with the primary amines present on the cell surface proteins to form stable thiourea bonds, which has solved the efflux problem observed with [^89^Zr]Zr-oxine^57–60^. The [^89^Zr]Zr-DFO-Bn-NCS has been successfully used to radiolabel mouse melanoma cells, mouse dendritic cells and human mesenchymal stem cells with insignificant efflux of free ^89^Zr from [^89^Zr]Zr-DFO-Bn-NCS over time (7 days-post radiolabeling)^58^. Additionally, a better version of the DFO chelator as DFO* has been developed to further strengthen the stability of ^89^Zr complexation and has shown lower bone uptake over time^61^. Various other chelators are also being developed to address in vivo stability of the ^89^Zr complex over time^62–66^.

**Figure 1:**
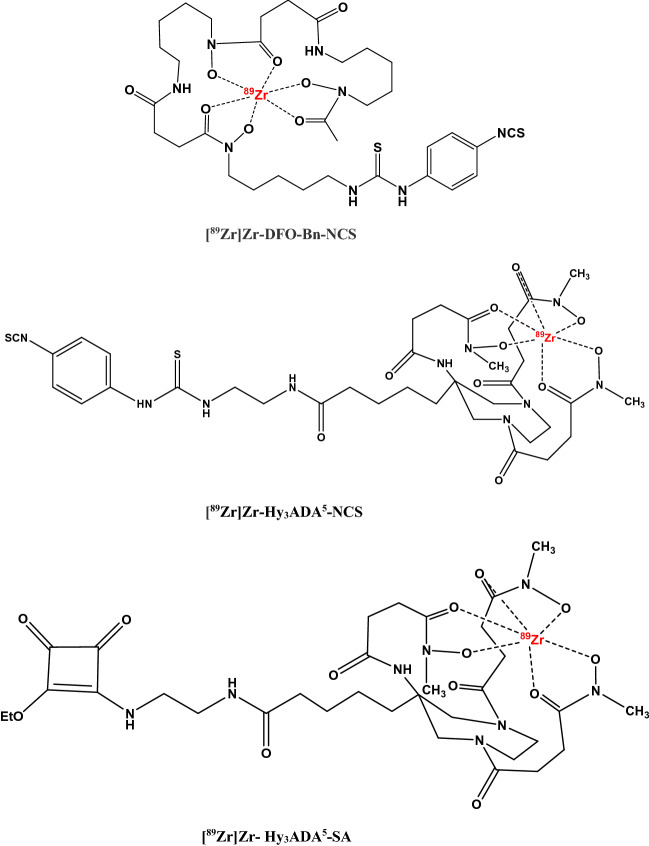
Chemical structures of [^89^Zr]Zr-DFO-Bn-NCS, [^89^Zr]Zr-Hy_3_ADA^5^-NCS and [^89^Zr]Zr- Hy_3_ADA^5^-SA.

In this work, we have optimized and compared the radiolabeling yields of WBCs and SCs using three different ready-to-use labeling synthons [^89^Zr]Zr-Hy_3_ADA^5^-NCS^66^, [^89^Zr]Zr-Hy_3_ADA^5^-SA^66^ and [^89^Zr]Zr-DFO-Bn-NCS^57–60^ (Fig. 1), and evaluated their applications in cell trafficking to better understand the biodistribution/pharmacokinetics of cell based therapies**.** This approach could be extended to various other cell-based therapies like CAR-T cell therapy.

## Results and discussion

### Production of [^89^Zr]ZrCl_4_ and radiosynthesis of [^89^Zr]Zr-DFO-Bn-NCS, [^89^Zr]Zr-Hy_3_ADA^5^-NCS and [^89^Zr]Zr-Hy_3_ADA^5^-SA

**Figure 2:**
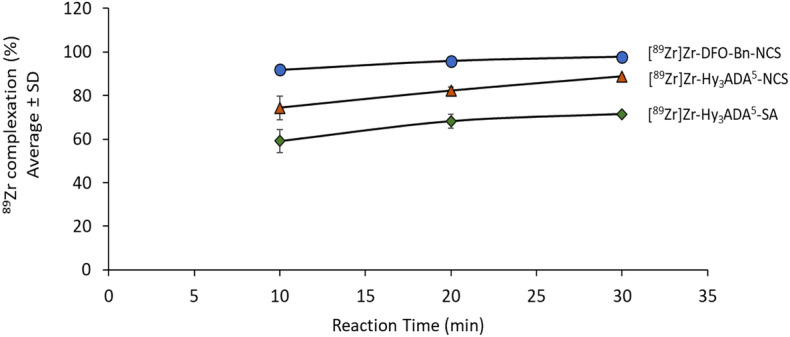
Percentage of ^89^Zr complexation with DFO-Bn-NCS, Hy_3_ADA^5^-NCS and Hy_3_ADA^5^-SA at different reaction times.

The PET isotope ^89^Zr was produced and purified in-house using a cyclotron as described earlier by Pandey et al.^67–70^ in a high apparent molar activity of 17.0–23.13 GBq/µmol, as assessed by complexing purified [^89^Zr]ZrCl_4_ with different amounts of DFO-Bn-NCS (Fig. S1, supplementary figure). All three synthons were successfully conjugated with ^89^Zr at 37 °C; pH 7.5–8.0 for 30 min in 72–98% radiolabeling yield. The DFO-Bn-NCS showed the highest complexation yield of 97.76 ± 0.31% (n = 3) followed by Hy_3_ADA^5^-NCS, 88.85 ± 0.05% (n = 3) and Hy_3_ADA^5^-SA, 71.58 ± 0.47% (n = 3) (Table 1, Fig. 2). These results indicate that acyclic chelator DFO-Bn-NCS imparts faster binding kinetics as compared to hybrid “cyclic-acyclic’ chelators Hy_3_ADA^5^-NCS and Hy_3_ADA^5^-SA. This is consistent with the complexation yield reported in our previous work, where a higher complexation yield was observed for DFO derivatives as compared to Hy_3_ADA derivatives^66^.

**Table 1:** Percentage of ^89^Zr complexation with DFO-Bn-NCS, Hy_3_ADA^5^-NCS and Hy_3_ADA^5^-SA at different reaction times.

Labeling condition	Reaction time	Percentage of ^89^Zr complexation with different synthons (average ± SD)		
		[^89^Zr]Zr-DFO-Bn-NCS (n = 3)	[^89^Zr]Zr-Hy_3_ADA^5^-NCS (n = 3)	[^89^Zr]Zr-Hy_3_ADA^5^-SA (n = 3)
37 °C, pH 7.5–8.0	10 min	91.83 ± 2.07	74.83 ± 5.38	59.08 ± 5.28
	20 min	95.89 ± 0.51	82.27 ± 1.53	68.27 ± 3.26
	30 min	97.76 ± 0.31	88.85 ± 0.05	71.58 ± 0.47

### Radiolabeling of WBCs and SCs

**Figure 3:**
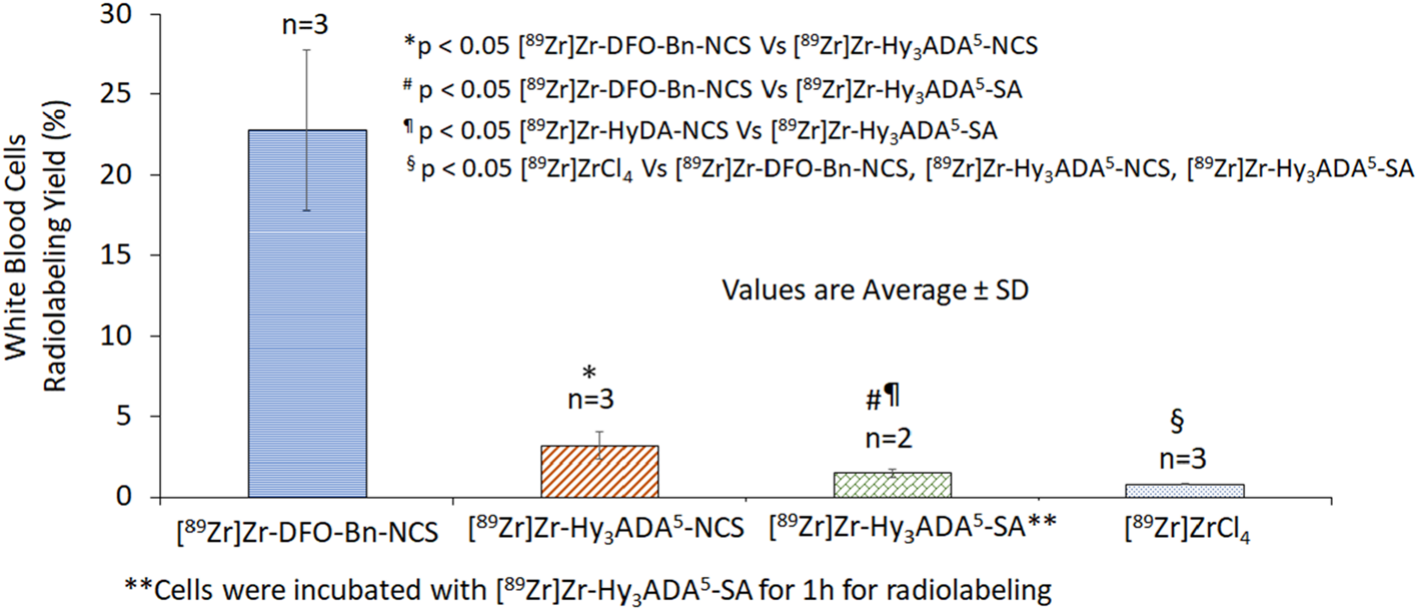
Radiolabeling of white blood cells (WBCs) with [^89^Zr]Zr-DFO-Bn-NCS, [^89^Zr]Zr-Hy_3_ADA^5^-NCS, [^89^Zr]Zr-Hy_3_ADA^5^-SA and [^89^Zr]ZrCl_4_.

**Figure 4:**
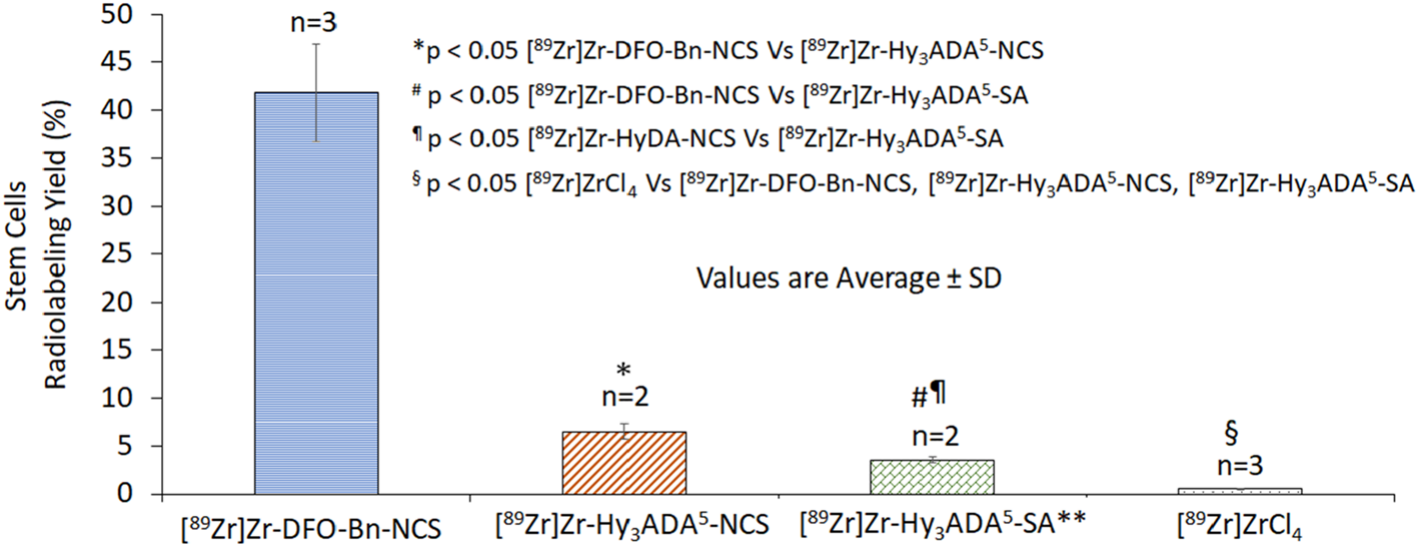
Radiolabeling of stem cells with [^89^Zr]Zr-DFO-Bn-NCS, [^89^Zr]Zr-Hy_3_ADA^5^-NCS, [^89^Zr]Zr-Hy_3_ADA^5^-SA and [^89^Zr]ZrCl_4_.

All three synthons were successfully employed to radiolabel WBCs and SCs, however their cell radiolabeling yield varied in the following order [^89^Zr]Zr-DFO-Bn-NCS > [^89^Zr]Zr-Hy_3_ADA^5^-NCS > [^89^Zr]Zr-Hy_3_ADA^5^-SA. For WBCs, the radiolabeling efficiency with [^89^Zr]Zr-DFO-Bn-NCS was 22.77 ± 5.02% (n = 3) as compared to 3.18 ± 0.86%; (n = 3) with [^89^Zr]Zr-Hy_3_ADA^5^-NCS and only 1.5 ± 0.28%; (n = 2) with [^89^Zr]Zr-Hy_3_ADA^5^-SA (Fig. 3). The radiolabeling efficiency for SCs with [^89^Zr]Zr-DFO-Bn-NCS was 41.83 ± 5.02% (n = 3) as compared to 6.57 ± 0.47% (n = 2) with [^89^Zr]Zr-Hy_3_ADA^5^-NCS and only 3.59 ± 0.27% (n = 2) with [^89^Zr]Zr-Hy_3_ADA^5^-SA (Fig. 4). The radiolabeled WBCs and SCs showed ~ 90–95% viability as per trypan blue exclusion cell viability assay. The difference in radiolabeling efficiencies between WBCs and SCs were expected due to the difference in their cell sizes and availability of surface proteins for conjugation and radiolabeling. The average cell size in the cell population was measured by TC10 cell counter (Biorad Laboratories, Inc. Hercules, CA) and found to be 4–10 µm for WBCs and 12–20 µm for SCs. Within a cell type, more cell labeling was observed with [^89^Zr]Zr-DFO-Bn-NCS as compared to [^89^Zr]Zr-Hy_3_ADA^5^-NCS and [^89^Zr]Zr-Hy_3_ADA^5^-SA. Further variation in radiolabeling yield could be explained by two aspects that may affect the cell radiolabeling yield. First, the degree of ^89^Zr-complexation with each chelator, where [^89^Zr]Zr-DFO-Bn-NCS had shown a relatively higher degree of ^89^Zr-complexation to begin with as compared to [^89^Zr]Zr-Hy_3_ADA^5^-NCS (~ 9% lower than [^89^Zr]Zr-DFO-Bn-NCS) and [^89^Zr]Zr-Hy_3_ADA^5^-SA (~ 27% lower than [^89^Zr]Zr-DFO-Bn-NCS). Second, the steric hindrance caused by hybrid chelators like Hy_3_ADA^5^-NCS and Hy_3_ADA^5^-SA during conjugation with cell surface proteins, suggesting a need to extend the length of the linkers in the case of Hy_3_ADA^5^-NCS and Hy_3_ADA^5^-SA chelators. The radiolabeling of cells with [^89^Zr]ZrCl_4_ as a negative control showed extremely poor radiolabeling efficiency for WBCs (0.80 ± 0.04%; n = 3) and stem cells (0.60 ± 0.01%; n = 3) as compared to radiolabeling efficiencies observed with the [^89^Zr]Zr-DFO-Bn-NCS, [^89^Zr]Zr-Hy_3_ADA^5^-NCS and [^89^Zr]Zr-Hy_3_ADA^5^-SA.

### Small animal PET imaging and biodistribution of ^89^Zr labeled WBCs

**Figure 5:**
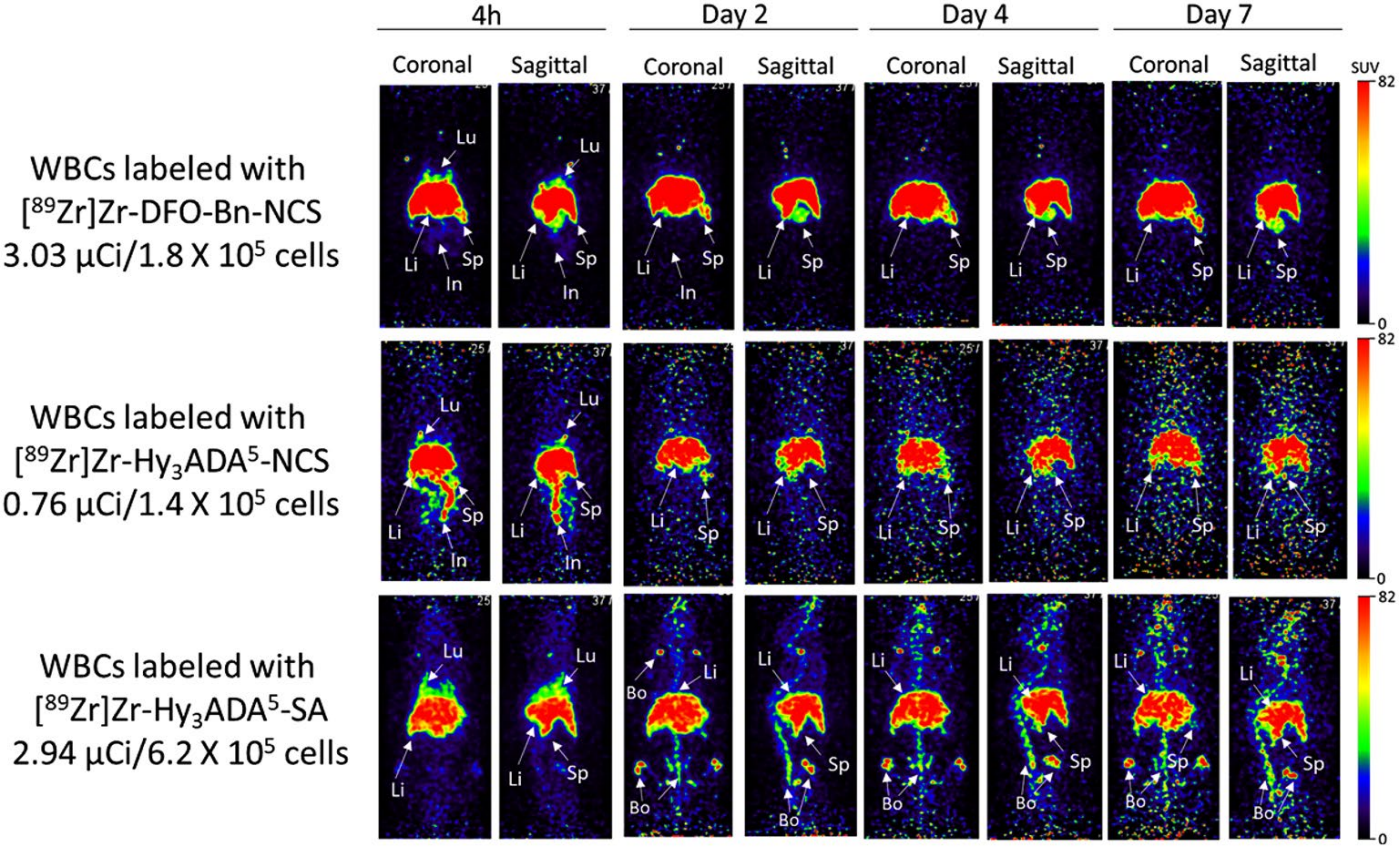
Representative coronal and sagittal PET maximum intensity projection (MIP) images showing distribution of WBCs labeled with [^89^Zr]Zr-DFO-NCS, [^89^Zr]Zr-Hy_3_ADA^5^-NCS and [^89^Zr]Zr-Hy_3_ADA^5^-SA synthons in athymic nude mice at different time points post-injection.

Small animal PET imaging and biodistribution of WBCs were performed independently after radiolabeling of WBCs with three different radiolabeling synthons, [^89^Zr]Zr-DFO-Bn-NCS, [^89^Zr]Zr-Hy_3_ADA^5^-NCS, and [^89^Zr]Zr-Hy_3_ADA^5^-SA to assess any variation in pharmacokinetics of WBCs radiolabeled with different synthons in healthy athymic mice (Fig. 5). After intravenous injection of WBCs-labeled with [^89^Zr]Zr-DFO-Bn-NCS, the majority of the radioactivity was observed in the liver and spleen, and remained significantly high at all time points (4 h, 2 days, 4 days, 7 days). Importantly, no bone uptake was observed at any time point over 7 days indicating good in vivo stability of [^89^Zr]Zr-WBCs radiolabeled with [^89^Zr]Zr-DFO-Bn-NCS. For WBCs radiolabeled with [^89^Zr]Zr-Hy_3_ADA^5^-NCS, significant localization of radioactivity was found in liver, spleen and intestine, a similar trend in distribution of radiolabeled WBCs as observed with [^89^Zr]Zr-DFO-Bn-NCS. Additionally, WBCs radiolabeled with [^89^Zr]Zr-Hy_3_ADA^5^-SA also showed primarily similar uptake in liver and spleen but increased uptake in bone over time suggesting instability of [^89^Zr]Zr-Hy_3_ADA^5^-SA conjugation with surface proteins or in vivo demetallation of ^89^Zr from [^89^Zr]Zr-Hy_3_ADA^5^-SA conjugate.

**Figure 6:**
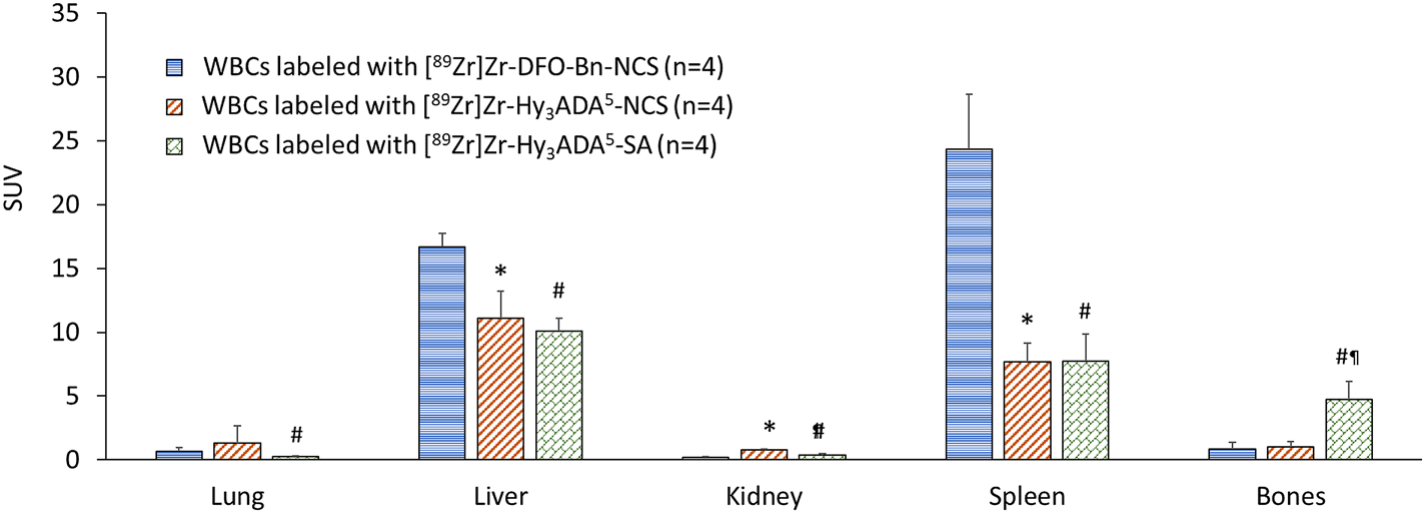
Uptake (SUV) and biodistribution of WBCs labeled with [^89^Zr]Zr-DFO-NCS, [^89^Zr]Zr-Hy_3_ADA^5^-NCS and [^89^Zr]Zr-Hy_3_ADA^5^-SA synthons in major organs of athymic nude mice at day 7 post-injection. *p < 0.05 WBCs labeled with [^89^Zr]Zr-DFO-Bn-NCS Vs [^89^Zr]Zr-Hy_3_ADA^5^-NCS; ^#^p < 0.05 WBCs labeled with [^89^Zr]Zr-DFO-Bn-NCS Vs [^89^Zr]Zr-Hy_3_ADA^5^-SA and ^¶^p < 0.05 WBCs labeled with [^89^Zr]Zr-Hy_3_ADA^5^-NCS Vs [^89^Zr]Zr-Hy_3_ADA^5^-SA.

**Figure 7:**
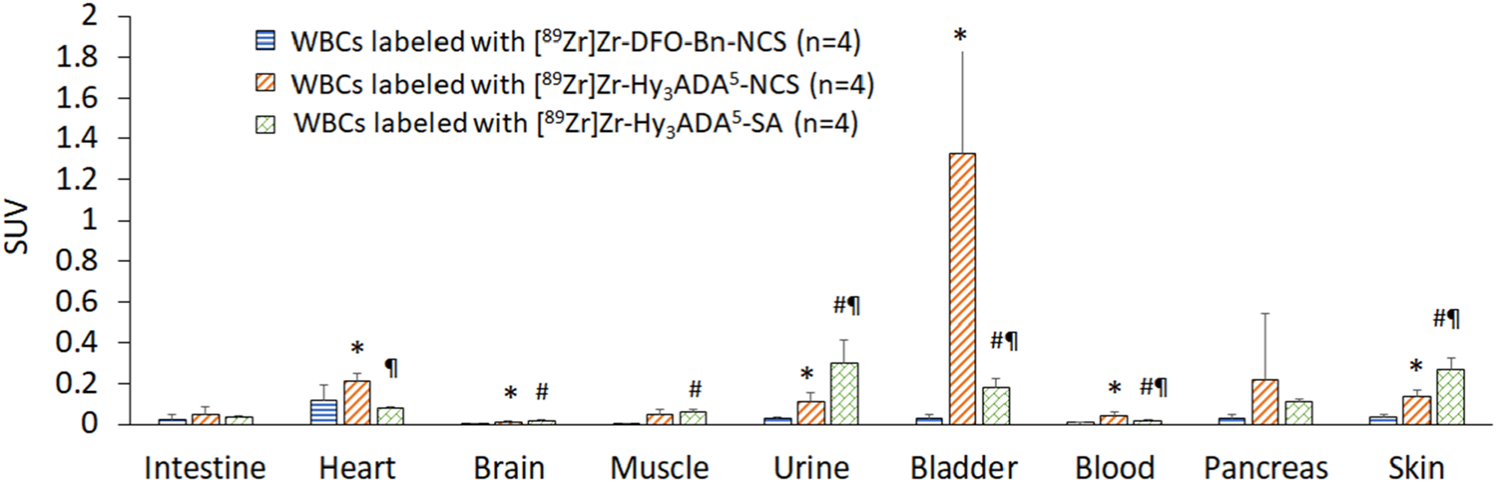
Uptake (SUV) and biodistribution of WBCs labeled with [^89^Zr]Zr-DFO-NCS, [^89^Zr]Zr-Hy_3_ADA^5^-NCS and [^89^Zr]Zr-Hy_3_ADA^5^-SA synthons in athymic nude mice at day 7 post-injection. *p < 0.05 WBCs labeled with [^89^Zr]Zr-DFO-Bn-NCS Vs [^89^Zr]Zr-Hy_3_ADA^5^-NCS; ^#^p < 0.05 WBCs labeled with [^89^Zr]Zr-DFO-Bn-NCS Vs [^89^Zr]Zr-Hy_3_ADA^5^-SA and ^¶^p < 0.05 WBCs labeled with [^89^Zr]Zr-Hy_3_ADA^5^-NCS Vs [^89^Zr]Zr-Hy_3_ADA^5^-SA.

Overall, the in vivo stability of [^89^Zr]Zr-DFO-Bn-NCS as demonstrated here is promising and superior over other synthons [^89^Zr]Zr-Hy_3_ADA^5^-NCS and [^89^Zr]Zr-Hy_3_ADA^5^-SA. Of these, [^89^Zr]Zr-Hy_3_ADA^5^-SA showed the lowest in-vivo stability but had considerably higher in vitro stability^66^. The biodistribution of radiolabeled WBCs in the rest of the major organs are presented in Figs. 6 and 7 and Table 2, indicating mild uptake in lung, heart, muscle, pancreas, and skin at 7 days post injection.

**Table 2:** Uptake (SUV) and biodistribution of white blood cells (WBCs) labeled with [^89^Zr]Zr-DFO-NCS (n = 4), [^89^Zr]Zr-Hy_3_ADA^5^-NCS (n = 4) and [^89^Zr]Zr-Hy_3_ADA^5^-SA (n = 4) synthons in athymic nude mice at day 7 post-injection.

Organ	White blood cells labeled with		
	[^89^Zr]Zr-DFO-Bn-NCS SUV average ± SD	[^89^Zr]Zr-Hy3ADA5-NCS SUV average ± SD	[^89^Zr]Zr-Hy3ADA5-SA SUV average ± SD
Lung	0.68 ± 0.26	1.32 ± 1.32	0.25 ± 0.056^#^
Liver	16.67 ± 1.05	11.12 ± 2.11*	10.08 ± 0.99^#^
Spleen	24.34 ± 4.30	7.69 ± 1.44	7.74 ± 2.10
Kidney	0.19 ± 0.75	0.76 ± 0.060*	0.37 ± 0.10^#^
Bones	0.83 ± 0.56	0.99 ± 0.42	4.70 ± 1.41^#¶^
Intestine	0.02 ± 0.02	0.046 ± 0.042	0.037 ± 0.006
Heart	0.12 ± 0.07	0.21 ± 0.038*	0.08 ± 0.003
Brain	0.003 ± 0.001	0.011 ± 0.006*	0.015 ± 0.0096^#^
Muscle	0.020 ± 0.027	0.049 ± 0.026	0.063 ± 0.0081^#^
Urine	0.030 ± 0.0044	0.11 ± 0.049*	0.30 ± 0.12^#¶^
Bladder	0.030 ± 0.017	1.33 ± 0.51*	0.18 ± 0.05^#¶^
Blood	0.0098 ± 0.0030	0.044 ± 0.019*	0.019 ± 0.006^#¶^
Pancreas	0.027 ± 0.023	0.22±0.32	0.11 ± 0.02^#^
Skin	0.036 ± 0.015	0.14 ± 0.034*	0.27 ± 0.05^#¶^

*p < 0.05 WBCs labeled with [^89^Zr]Zr-DFO-Bn-NCS Vs [^89^Zr]Zr-Hy_3_ADA^5^-NCS.

^#^p < 0.05 WBCs labeled with [^89^Zr]Zr-DFO-Bn-NCS Vs [^89^Zr]Zr-Hy_3_ADA^5^-SA.

^¶^p < 0.05 WBCs labeled with [^89^Zr]Zr-Hy_3_ADA^5^-NCS Vs [^89^Zr]Zr-Hy_3_ADA^5^-SA.

### Small animal PET imaging and biodistribution of ^89^Zr labeled SCs

**Figure 8:**
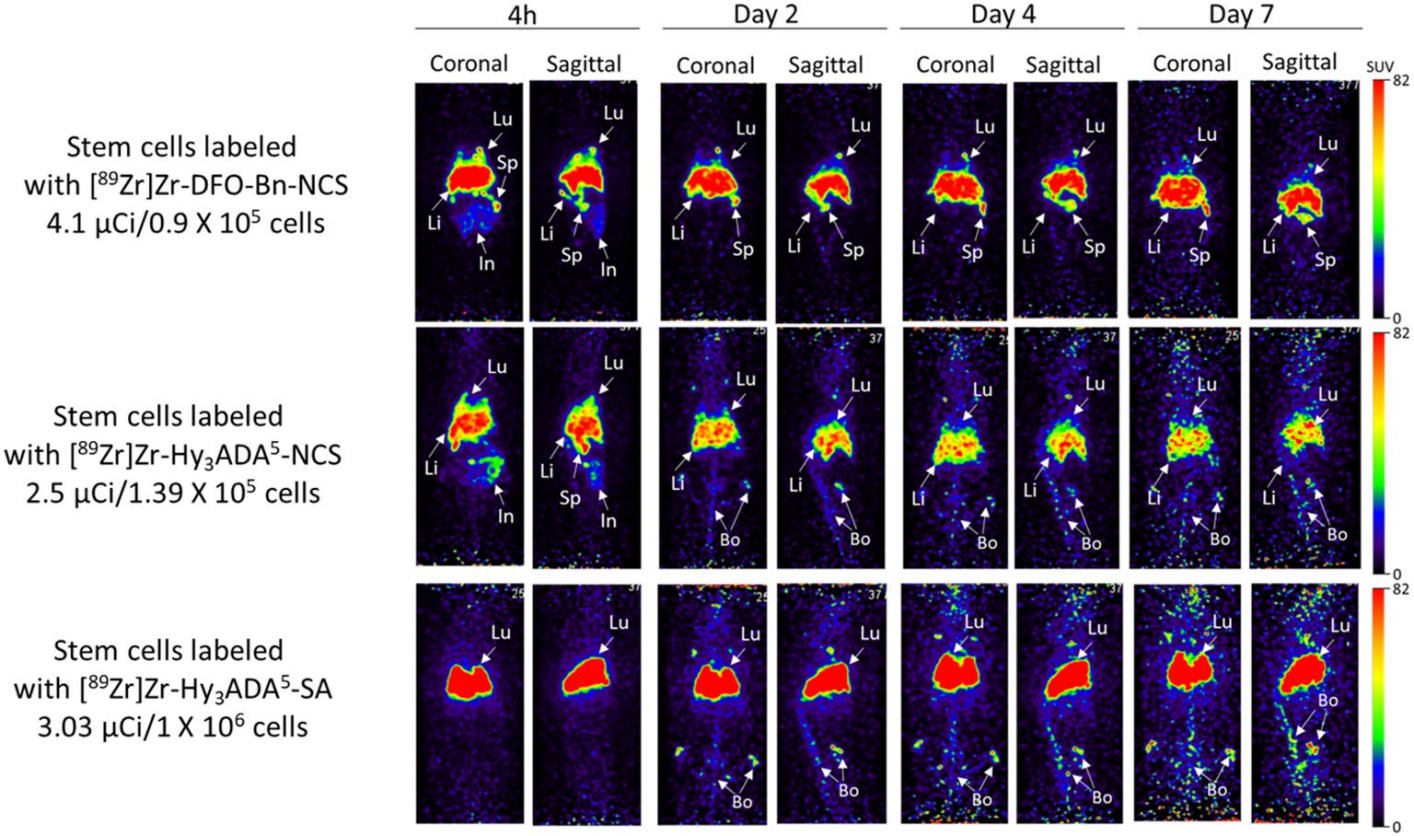
Representative coronal and sagittal PET maximum intensity projection (MIP) images showing distribution of stem cells labeled with [^89^Zr]Zr-DFO-NCS, [^89^Zr]Zr-Hy_3_ADA^5^-NCS and [^89^Zr]Zr-Hy_3_ADA^5^-SA synthons in athymic nude mice at different time points post-injection.

**Figure 9:**
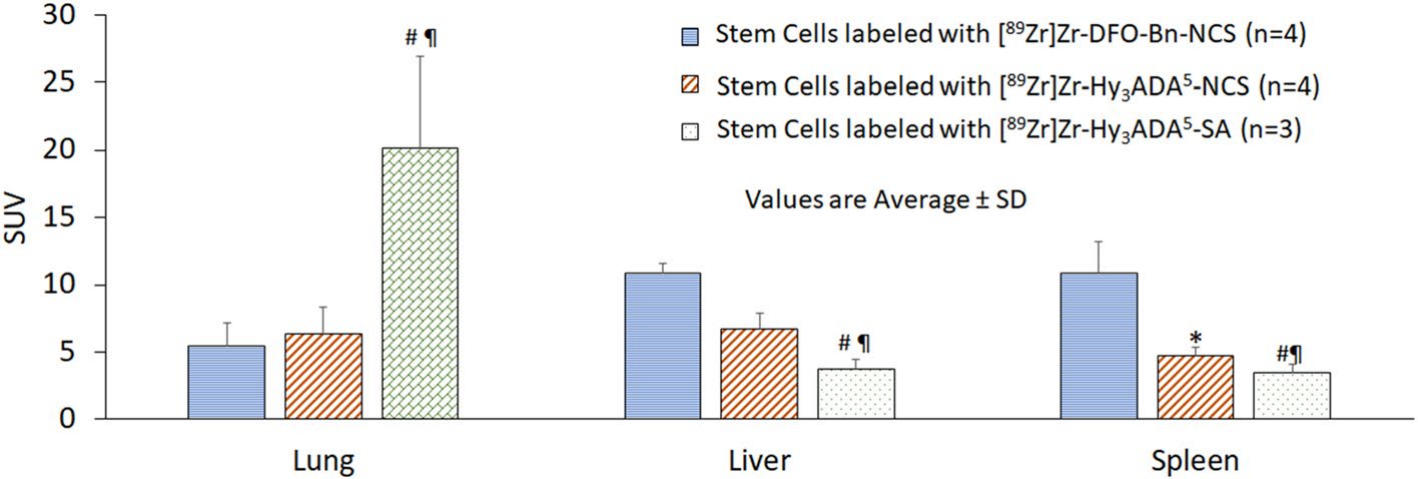
Uptake (SUV) and biodistribution of stem cells labeled with [^89^Zr]Zr-DFO-NCS, [^89^Zr]Zr-Hy_3_ADA^5^-NCS and [^89^Zr]Zr-Hy_3_ADA^5^-SA synthons in lung, liver and spleen of athymic nude mice at day 7 post-injection. *p < 0.05 stem cells labeled with [^89^Zr]Zr-DFO-Bn-NCS Vs with [^89^Zr]Zr-Hy_3_ADA^5^-NCS; ^#^p < 0.05 stem cells labeled with [^89^Zr]Zr-DFO-Bn-NCS Vs [^89^Zr]Zr-Hy_3_ADA^5^-SA and ^¶^p < 0.05 stem cells labeled with [^89^Zr]Zr-Hy_3_ADA^5^-NCS Vs [^89^Zr]Zr-Hy_3_ADA^5^-SA.

**Figure 10:**
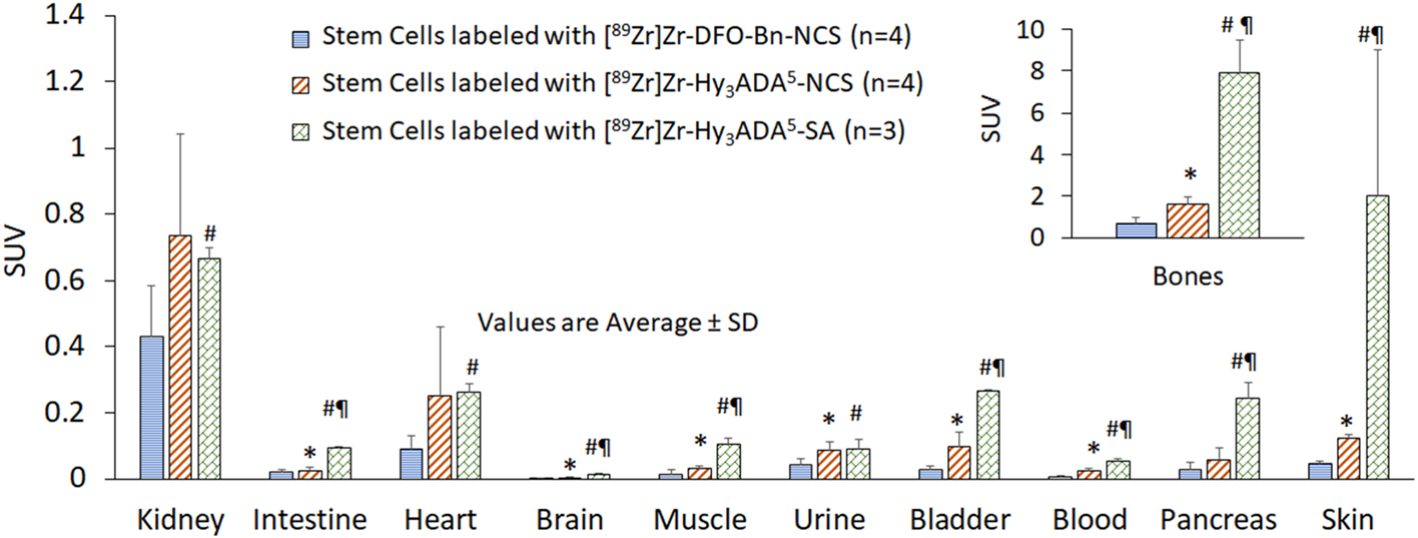
Uptake (SUV) and biodistribution of stem cells labeled with [^89^Zr]Zr-DFO-NCS, [^89^Zr]Zr-Hy_3_ADA^5^-NCS and [^89^Zr]Zr-Hy_3_ADA^5^-SA synthons in athymic nude mice at day 7 post-injection. *p < 0.05 stem cells labeled with [^89^Zr]Zr-DFO-Bn-NCS Vs [^89^Zr]Zr-Hy_3_ADA^5^-NCS; ^#^p < 0.05 stem cells labeled with [^89^Zr]Zr-DFO-Bn-NCS Vs [^89^Zr]Zr-Hy_3_ADA^5^-SA and ^¶^p < 0.05 stem cells labeled with [^89^Zr]Zr-Hy_3_ADA^5^-NCS Vs [^89^Zr]Zr-Hy_3_ADA^5^-SA.

Small animal PET imaging and biodistribution of SCs were performed independently after radiolabeling of SCs with three different radiolabeling synthons, [^89^Zr]Zr-DFO-Bn-NCS, [^89^Zr]Zr-Hy_3_ADA^5^-NCS, and [^89^Zr]Zr-Hy_3_ADA^5^-SA to assess any variation in pharmacokinetics of SCs radiolabeled with different synthons in healthy athymic mice (Fig. 8). The SCs radiolabeled with ^89^Zr using [^89^Zr]Zr-DFO-Bn-NCS and [^89^Zr]Zr-Hy_3_ADA^5^-NCS showed uptake primarily in lung, liver and spleen at all time points with some early uptake in intestine. The SCs radiolabeled with [^89^Zr]Zr-Hy_3_ADA^5^-NCS showed some localization of radioactivity in lung, liver, spleen and intestine but had prominent accumulation of radioactivity in bones at day 2 post-injection. The detection of radioactivity signals in bone of SCs radiolabeled with [^89^Zr]Zr-Hy_3_ADA^5^-NCS but not with [^89^Zr]Zr-DFO-Bn-NCS suggested in vivo instability of [^89^Zr]Zr-Hy_3_ADA^5^-NCS conjugate with surface proteins. Interestingly, the small animal PET imaging of SCs radiolabeled with [^89^Zr]Zr-Hy_3_ADA^5^-SA showed most of the radioactivity in lungs at all time points, whereas the radioactivity in bones significantly increased from day 2 onwards. The significant increase in radioactivity accumulation in bone further suggested in vivo instability of [^89^Zr]Zr-Hy_3_ADA^5^-SA. Overall, synthon [^89^Zr]Zr-DFO-Bn-NCS was found to be a superior performer in stem cell radiolabeling and tracking over time as compared to [^89^Zr]Zr-Hy_3_ADA^5^-NCS and [^89^Zr]Zr-Hy_3_ADA^5^-SA. The biodistribution of radiolabeled SCs in the rest of the major organs are presented in Figs. 9 and 10 and Table 3, indicating mild uptake in lung, heart, kidney, muscle, pancreas, and skin at 7 days post injection.

**Table 3:** Uptake (SUV) and biodistribution of stem cells labeled with [^89^Zr]Zr-DFO-NCS (n = 4) , [^89^Zr]Zr-Hy_3_ADA^5^-NCS (n = 4) and [^89^Zr]Zr-Hy_3_ADA^5^-SA (n = 3) synthons in athymic nude mice at day 7 post-injection.

Organ	Stem cells labeled with		
	[^89^Zr]Zr-DFO-Bn-NCS	[^89^Zr]Zr-Hy_3_ADA^5^-NCS	[^89^Zr]Zr-Hy_3_ADA^5^-SA
	SUV average ± SD	SUV average ± SD	SUV average ± SD
Lung	5.44 ± 1.72	6.35 ± 1.98	20.15 ± 6.79^#¶^
Liver	10.88 ± 0.72	6.75 ± 1.17	3.73 ± 0.69^#¶^
Spleen	10.88 ± 2.37	4.69 ± 0.69*	3.44 ± 0.61^#¶^
Kidney	0.43 ± 0.15	0.74 ± 0.30	0.67 ± 0.03^#^
Bones	0.68 ± 0.27	1.61 ± 0.36*	7.91 ± 1.55^#¶^
Intestine	0.022 ± 0.006	0.024 ± 0.010	0.09 ± 0.004
Heart	0.09 ± 0.04	0.25 ± 0.21	0.26 ± 0.02
Brain	0.002 ± 0.001	0.004 ± 0.001	0.012 ± 0.004^#^
Muscle	0.014 ± 0.013	0.033 ± 0.005*	0.11 ± 0.018^#¶^
Urine	0.044 ± 0.018	0.086 ± 0.024*	0.092 ± 0.028^#¶^
Bladder	0.029 ± 0.01	0.099 ± 0.041*	0.27 ± 0.002^#¶^
Blood	0.0072 ± 0.0025	0.02 ± 0.006*	0.055 ± 0.006^#¶^
Pancreas	0.029 ± 0.020	0.058 ± 0.036	0.24 ± 0.048^¶^
Skin	0.047 ± 0.006	0.12 ± 0.013*	0.86 ± 0.44^#¶^

*p < 0.05 stem cells labeled with [^89^Zr]Zr-DFO-Bn-NCS Vs [^89^Zr]Zr-Hy_3_ADA^5^-NCS; ^#^p < 0.05 stem cells labeled with [^89^Zr]Zr-DFO-Bn-NCS Vs [^89^Zr]Zr-Hy_3_ADA^5^-SA and ^¶^p < 0.05 stem cells labeled with [^89^Zr]Zr-Hy_3_ADA^5^-NCS Vs [^89^Zr]Zr-Hy_3_ADA^5^-SA.

### Small animal PET imaging of un-chelated [^89^Zr]ZrCl_4_

**Figure 11:**
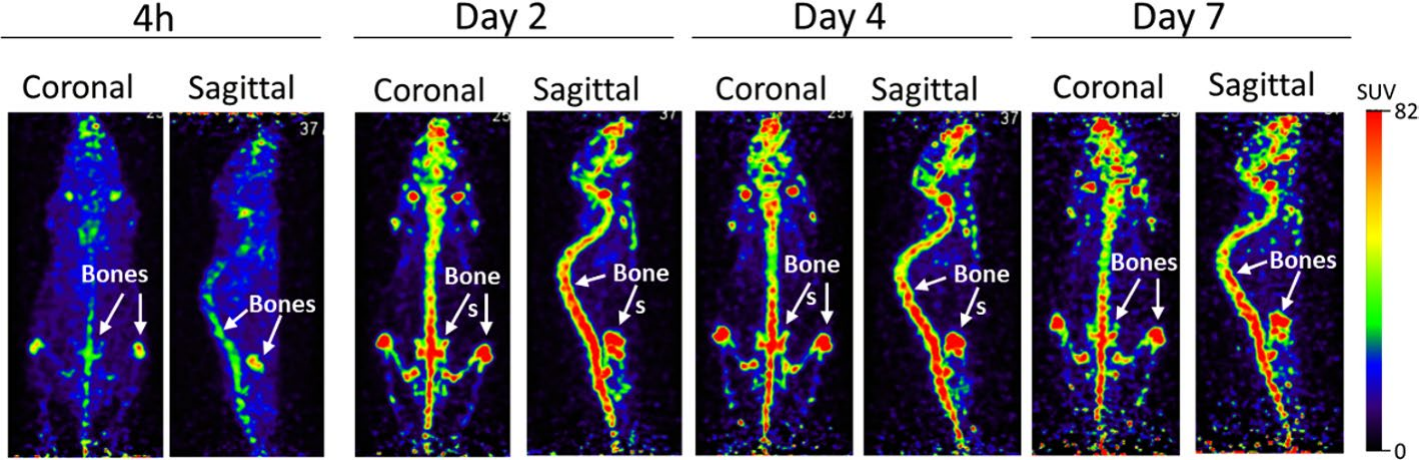
Representative coronal and sagittal PET maximum intensity projection (MIP) images showing distribution of [^89^Zr]ZrCl_4_ in athymic nude mice at different time points post-injection.

**Figure 12:**
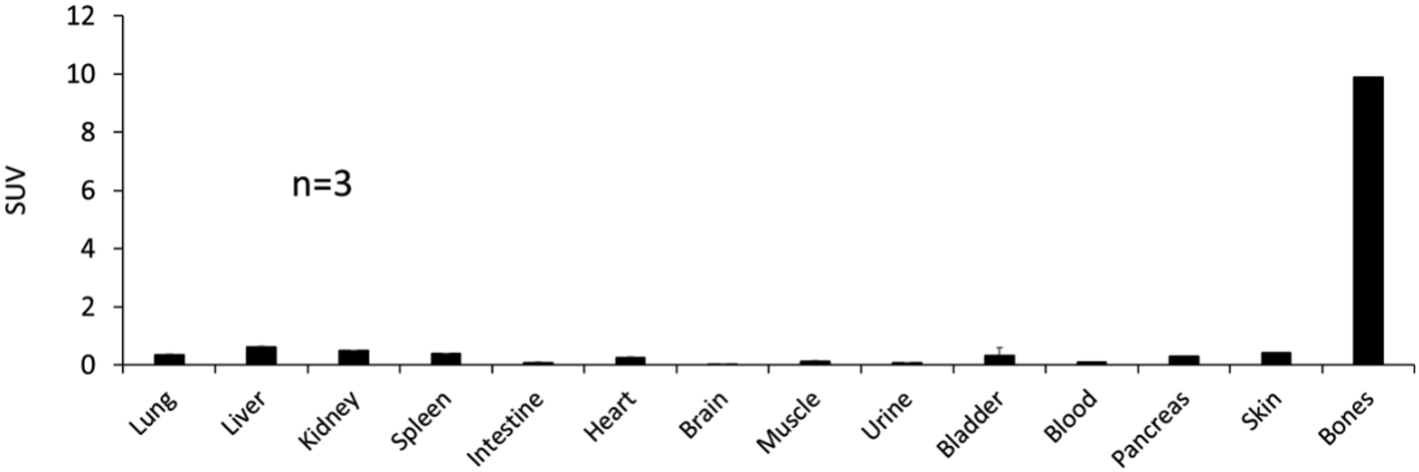
Uptake (SUV) and distribution of [^89^Zr]ZrCl_4_ in athymic nude mice at day 7 post-injection.

The in vivo characteristics of un-chelated [^89^Zr]ZrCl_4_ was also investigated (Fig. 11). The small animal PET imaging showed a high accumulation of free ^89^Zr in the bones at 4 h and did not distribute to the lung, liver, spleen, or any other organs at any other time points as were noted with radiolabeled WBCs and SCs. The radioactivity increased significantly on day 2 and remained in the bones until day 7, attributed to the entrapment of osteophilic ^89^Zr and its poor clearance from the bones (Fig. 12, Table 4). This observation is consistent with findings by Abou et al. 2011 demonstrating that [^89^Zr]ZrCl_4_ is a bone-seeking species and accumulates in bones and joints post-administration in mice^71^.

**Table 4:** Uptake (SUV) and distribution of [^89^Zr]ZrCl_4_ in athymic nude mice (n = 3) at day 7 post-injection.

Free Zr-89	SUV average ± SD
Lung	0.36 ± 0.06
Liver	0.61 ± 0.07
Kidney	0.49 ± 0.07
Spleen	0.39 ± 0.09
Intestine	0.09 ± 0.01
Heart	0.26 ± 0.03
Brain	0.02 ± 0.02
Muscle	0.14 ± 0.05
Urine	0.08 ± 0.03
Bladder	0.32 ± 0.09
Blood	0.12 ± 0.01
Pancreas	0.29 ± 0.02
Skin	0.42 ± 0.14
Bones	9.89 ± 0.4

## Conclusion

Both WBCs and SCs were successfully directly radiolabeled with ^89^Zr using three different ready-to-use synthons, [^89^Zr]Zr-DFO-Bn-NCS, [^89^Zr]Zr-Hy_3_ADA^5^-NCS and [^89^Zr]Zr-Hy_3_ADA^5^-SA. The radiolabeling efficiencies of cells (WBCs and SCs) were significantly higher with [^89^Zr]Zr-DFO-Bn-NCS than ^89^Zr]Zr-Hy_3_ADA^5^-NCS and [^89^Zr]Zr-Hy_3_ADA^5^-SA. The higher cell radiolabeling efficiency with [^89^Zr]Zr-DFO-Bn-NCS could be attributed to an open chain structure of DFO. In vivo, the stability of ^89^Zr complexed with DFO and Hy_3_ADA^5^ chelators were found to be comparable. The synthons [^89^Zr]Zr-DFO-Bn-NCS, and [^89^Zr]Zr-Hy_3_ADA^5^-NCS could be considered to radiolabel cells for further application; synthon [^89^Zr]Zr-Hy_3_ADA^5^-SA showed higher bone uptake indicating its poor stability (synthon-protein conjugation) in vivo. Further optimization of the [^89^Zr]Zr-Hy_3_ADA^5^-SA synthon is needed to enhance cell radiolabeling efficiency and stability. Overall, the PET-based cell radiolabeling methodology offers an effective tool to noninvasively track WBCs, SCs, and other cells to understand the safety, efficacy, distribution, and clearance of cell-based therapies.

## Materials and methods

### General

The ^89^Zr used in this study was produced on a PETtrace cyclotron (GE Healthcare, Waukesha, WI) using ^89^Y target foil (0.1 mm; 50 X 50 mm, 99.9%), which was purchased from Alfa-Aesar, Haverhill, MA. The trace metal grade nitric acid (67–70%) and hydrochloric acid (34–37%) were purchased from Thermo Fisher Scientific, Waltham, MA. Sodium bicarbonate, oxalic acid dehydrate (*Trace*SELECT^®^ ≥ 99.9999% metal basis), sodium carbonate, sodium citrate dihydrate and HPLC grade acetonitrile were purchased from Sigma Aldrich, St. Louis, MO. The silica gel iTLC was purchased from Agilent Technologies, Santa Clara, CA. The chelator p-SCN-Bn-Deferoxamine or DFO-Bn-NCS (≥ 94%) was purchased from Macrocylics, Plano, TX, whereas the other two chelators Hy_3_ADA^5^-NCS and Hy_3_ADA^5^-SA were synthesized as described by Klasen et al.^66^. The empty Luer-Inlet SPE cartridges (1 mL) with frits (20 µm pore size) were purchased from Supelco Inc (Bellefonte, PA) and Chromafix^®^ 30-PS-HCO_3_ PP cartridges (45 mg) were purchased from Macherey–Nagel, Duren, Germany. The Millex ^®^-GV filter (0.2 µm) was purchased from Millipore Sigma, Burlington, MA. The hydroxamate resin was synthesized in-house as demonstrated by Pandey et al.^67–70^ The Thermomixer was purchased from Eppendorf, Hamburg, Germany.

### Production and purification of [^89^Zr]ZrCl_4_

The ^89^Zr was produced using yttrium foil on a solid target through a ^89^Y(p,n)^89^Zr nuclear reaction in a PETtrace cyclotron as described previously by Pandey et al.^68^. ^89^Zr was purified first as [^89^Zr]Zr-oxalate and then converted to [^89^Zr]ZrCl_4_ using activated Chromafix 30-PS-HCO_3_ SPE as demonstrated by Pandey^67–70^ and Larenkov et al.^72^, respectively. The final [^89^Zr]ZrCl_4_ was eluted in ⁓ 0.5 mL of 1.0 N HCl and then dried using a steady flow of nitrogen gas in a V-vial at 65 °C.

### Apparent molar activity of [^89^Zr]ZrCl_4_

The apparent molar activity of ^89^Zr was estimated using a DFO-Bn-NCS titration method. In this method, 10µL [^89^Zr]ZrCl_4_ (36.4 MBq) was added to 90µL de-ionized H_2_O. To this, 4 µL of 0.5 M Na_2_CO_3_ was added to neutralize and adjust the pH to 7.5–8.0. To the neutralized mixture, 0.01–10 µg of DFO-Bn-NCS in 4µL of DMSO was added and mixed. The complexation mixture was then incubated at 37 °C for 1 h. After 1 h, the degree of ^89^Zr complexation was determined with respect to the DFO-Bn-NCS concentration using radio-TLC with 20 mM sodium citrate (pH 4.9–5.1) as a mobile phase. The complexed ^89^Zr as [^89^Zr]Zr-DFO-Bn-NCS showed at the origin with R_f_ = 0, whereas free or un-complexed ^89^Zr had an R_f_ of 0.99 (solvent front). The half maximal inhibitory concentration (IC_50_) of DFO-Bn-NCS in mg/mL was calculated using non-linear ﻿regression curve fitting analysis.

The analysis was performed using analysis software—GraphPad Prism 9 (GraphPad Software, San Diego, CA). The minimum ligand concentration for which 100% complexation occurred was estimated by multiplying the IC_50_ by 2, and the apparent molar activity (GBq/µmole) and the apparent specific activity (GBq/mg) of ^89^Zr were calculated by correcting for the total activity divided by µmoles or mg of DFO-Bn-NCS needed for 100% ^89^Zr complexation.

### Radiosynthesis of [^89^Zr]Zr-DFO-Bn-NCS, [^89^Zr]Zr-Hy_3_ADA^5^-NCS and [^89^Zr]Zr-Hy_3_ADA^5^-SA

The radiosynthesis of the different synthons [^89^Zr]Zr-DFO-Bn-NCS, [^89^Zr]Zr-Hy_3_ADA^5^-NCS and [^89^Zr]Zr-Hy_3_ADA^5^-SA were performed using a modified procedure demonstrated in our previous work.^58^ The purified [^89^Zr]ZrCl_4_ was resuspended in appropriate volume of 0.1 N HCl and then neutralized to pH ⁓ 8.0 with 0.5 M Na_2_CO_3._ The neutralized [^89^Zr]ZrCl_4_ solution (70 -100 µL) containing ~ 21 MBq of ^89^Zr was used in the case of DFO-Bn-NCS, whereas ~ 61 to 68 MBq of ^89^Zr was used in the case of Hy_3_ADA^5^-NCS and Hy_3_ADA^5^-SA. To this neutralized [^89^Zr]ZrCl_4_ solution, 4 nmoles of DFO-Bn-NCS or Hy_3_ADA^5^-NCS or Hy_3_ADA^5^-SA (prepared in DMSO) were added in separate reactions. The resultant reaction mixtures were stirred for 30 min at 37 °C in a thermomixer at 500 rpm. The radiolabeling efficiency was determined at different time points by silica radio-TLC using 20 mM sodium citrate (pH 4.9–5.1) as a mobile phase.

### Cell preparation

The human mesenchymal SCs were gifted by Dr. Atta Behfar from the Department of Cardiovascular Medicine, Mayo Clinic, Rochester, MN, USA, and WBCs were isolated from the peripheral blood provided by the Division of Transfusion Medicine, Mayo Clinic, Rochester, MN, USA. The isolation of WBCs from the peripheral blood was performed using Lymphoprep™ (STEMCELL Technologies Inc., Canada) gradient centrifugation method as per manufacturer instructions. The final WBC solution was washed with Hank’s Balanced Salt Solution.

### Cell radiolabeling

The SCs and WBCs cells were radiolabeled with different synthons, [^89^Zr]Zr-DFO-Bn-NCS, [^89^Zr]Zr-Hy_3_ADA^5^-NCS and [^89^Zr]Zr-Hy_3_ADA^5^-SA separately. The cell radiolabeling mixture was prepared by mixing equal volume of the [^89^Zr]Zr-DFO-Bn-NCS or [^89^Zr]Zr-Hy^3^ADA^5^-NCS or [^89^Zr]Zr-Hy_3_ADA^5^-SA reaction mix and equal volume of phosphate buffer-HEPES. The phosphate buffer-HEPES was prepared by mixing 120 µL of 1.2 M phosphate buffer and 100 µL of 1 M HEPES. This cell radiolabeling mix was incubated at room temperature for 30 min. As a negative control, a cell radiolabeling mix with [^89^Zr]ZrCl_4_ was also tested for radiolabeling SCs and WBCs. After this incubation, the cell radiolabeling mixture (~ 150 to 200 µL) was added to a cell suspension at a concentration of 6 × 10^6^ cells in 500 µL HEPES Buffered Hank’s Balanced Salt Solution at pH 7.5–8.0^58,59^. The cell radiolabeling was performed for 30 min at room temperature for WBCs and 37 °C for SCs in a thermomixer. After radiolabeling, the cells were washed 3× with complete Dulbecco's Modified Eagle Medium.

### Trypan blue exclusion assay cellular viability test

The effect of radiolabeling on cellular viability was assessed using the trypan blue exclusion assay test within 1 h of labeling.

#### Animals

8–10 week old athymic nude mice (male and female, 1:1) were obtained from Charles Rivers Laboratories or Taconic Biosciences, Inc.

### PET imaging and ex vivo biodistribution studies

After radiolabeling, the radiolabeled WBCs (0.1–0.6 × 10^6^; 0.03–0.11 MBq); and SCs (0.1–1 × 10^6^; 0.1–0.15 MBq) were injected via tail vein into a group (n = 3) of athymic nude mice. PET images were acquired at 4 h, 2 days, 4 days and 7 days post-injection (p.i.) using a small animal PET scanner. The free [^89^Zr]ZrCl_4_ with radioactivity (0.15–0.19 MBq) was also injected intravenously via the tail vein. The small animal PET images were visualized, analyzed, and scaled to SUV using image analysis software, MIM 7 software (MIM Software Inc., Cleveland, OH, USA). The PET images are shown as maximum intensity projection (MIP) images in the coronal and sagittal plane. The animals were euthanized at 7d p.i., and organs/tissues collected to measure the standardized uptake value (SUV) in major organs. Animals were euthanized via cardiectomy under anesthesia using isoflurane as approved by the Institutional Animal Care and Use Committee (IACUC) of the Mayo Clinic Rochester MN USA. SUV was calculated using following formula:

$$Standardized \,Uptake\, Value =\frac{Radioactivity \,concentration\, in\, tissue\, (\upmu Ci/g)}{{\frac{Injected \,dose\, (\upmu Ci)}{Body\, Weight (g)}}}$$

### Statistics

The obtained data were analyzed using Microsoft Excel program and the results were compared using unpaired Student’s t-test analysis. Differences were regarded as statistically significant for p < 0.05.

### Ethical standards

Studies were conducted with proper use and care of animals as approved by the Institutional Animal Care and Use Committee of the Mayo Clinic Rochester MN USA. Additionally, all methods were also performed in accordance with Institutional Animal Care and Use Committee’s guidelines and regulation. These guidelines are equivalent to the ARRIVE guidelines and therefore all methods were also performed in accordance with ARRIVE guidelines.

## Supplementary Information

Supplementary Figure S1.

## Data Availability

All data generated or analyzed during this study are included in this published article.
