# Supplementary material for: Evaluation of different 89Zr-labeled synthons for direct labeling and tracking of white blood cells and stem cells in healthy athymic mice
Source: Sci Rep. 2022 Sep 19;12:15646. doi: 10.1038/s41598-022-19953-4 (PMC9485227; doi:10.1038/s41598-022-19953-4)
Supplement: Supplementary file 1 — Supplementary Figure S1. [file 41598_2022_19953_MOESM1_ESM.docx]

**Supplementary Information**

**Evaluation of different ^89^Zr-labeled synthons for direct labeling and tracking of white blood cells and stem cells in healthy athymic mice**

Aditya Bansal^a^, Shalini Sharma^a^, Benedikt Klasen^b^, Frank Rosch^b^, Mukesh K. Pandey^a^*

^a^ Division of Nuclear Medicine, Department of Radiology, Mayo Clinic, Rochester, MN, 55906, USA

^b^ Department of Chemistry-TRIGA site, Johannes Gutenberg University Mainz, Germany

**Figure S1.** Non-Linear Regression Fit Curves of DFO-Bn-NCS and ^89^Zr complexation – A. [Concentration] vs Complexation (three parameter) and B. log (concentration) vs. complexation (three parameter) for calculating IC_50_ of DFO-Bn-NCS used in the determination of apparent molar activity (Am) of a purified [^89^Zr]ZrCl_4_ solution.

**
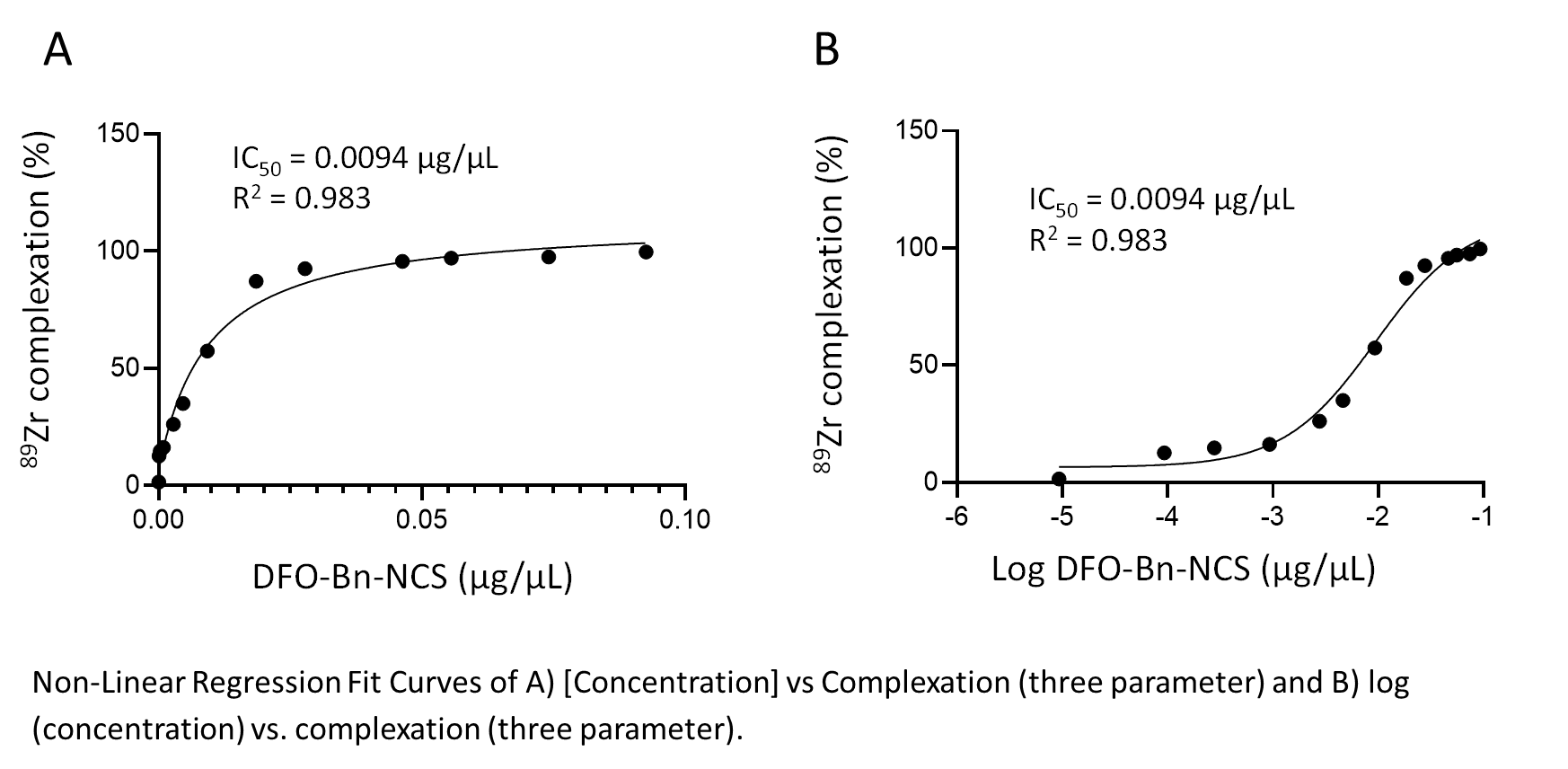
**
